# Resilience and mental health among juveniles: role of strategies for coping with stress

**DOI:** 10.1186/s12955-021-01701-3

**Published:** 2021-02-18

**Authors:** Karol Konaszewski, Małgorzata Niesiobędzka, Janusz Surzykiewicz

**Affiliations:** 1 Institute of Psychology, Polish Academy of Sciences, Warsaw, Poland; 2 Faculty of Education, University of Bialystok, Bialystok, Poland; 3 Faculty of Philosophy and Education, Katholische Universität Eichstätt-Ingolstadt, Eichstätt, Germany; 4 Faculty of Education, Cardinal Stefan Wyszynski University in Warsaw, Warsaw, Poland

## Abstract

**Background:**

Mental health is an important aspect of the process of individual adaptation and development. The present study analysed the role played by resilience in mental health while taking into account both positive and negative indicators among juveniles. The aim of the first study (Study 1) is to analyse the relationship between resilience and the broadly understood mental health of juveniles admitted to youth education centres. Study 2 aimed to understand the direct and indirect role of resilience in shaping the mental health of juveniles. In the model we tested, we looked at the relationship among resilience, coping strategies, and mental well-being.

**Methods:**

The first study involved 201 juveniles, and the second involved 253 juveniles. Resilience was measured by the Resilience Scale-14. Coping strategies were measured with the Brief-COPE Questionnaire, and information on mental health was obtained using the Kutcher Adolescent Depression Scale, the Satisfaction with Life Scale and the Warwick-Edinburgh Mental Well-being Scale.

**Results:**

The results of both studies have shown that resilience is an important predictor of the mental health of juveniles, primarily with respect to its positive indicator. The stronger the severity of resilience, the greater the satisfaction with life and mental well-being of the juveniles surveyed. In addition, two coping strategies (seeking support from others and coping through emotions) mediated the relationship between resilience and mental well-being.

**Conclusions:**

The results obtained indicate that work at the level of juvenile resilience leads to the strengthening of positive mental health indicators and buffering of negative indicators.

## Introduction

Mental health is an important aspect of the process of individual adaptation and development. Traditionally, the concept of health is based on a disease model and is defined as the absence of pain, disorder or disease. Research concerning this approach focuses on how to reduce or eliminate negative mental health conditions such as maladaptation, depression and suicidal thoughts. However, the concept of health in recent years includes not only the absence of a negative state but also the emergence of a positive state [1, 2]. The proposed two-dimensional mental health model has provided a solid basis for a more comprehensive and accurate diagnosis of individual health indicators [3, 4]. Within this model, health is considered to be a complete condition whose meaning is greater than an absence of illness or a sense of subjective well-being [5–7]. Therefore, the measurement and assessment of mental health should include both negative and positive indicators [8, 9]. For the present study, it was decided to use both types of mental health indicators. According to the Grand Challenges in the Global Mental Health Initiative [10], in addition to both types of mental health indicators, personal resources should be identified as a means of helping individuals overcome difficulties and adjust to circumstances [11]. For these reasons, the present study analysed the role played by resilience in mental health while taking into account both positive and negative indicators.

Generally, young people experience different psychological, emotional and behavioural challenges or problems, especially when they are near the boundaries between childhood, adolescence and youth. Multidimensional barriers or life events could put them at risk of developing a sense of dissatisfaction with life that can lead to negative health outcomes, making them a vulnerable part of the young population. For this reason, research on mental health and well-being has been growing in recent decades. Worldwide, a significant percentage of adolescents experience mental health problems [12–18]. The World Health Organization places particular emphasis on the mental health of teenagers, noting that more than half of all mental illnesses begin during adolescence [7, 19]. One of these illnesses is depressive disorder, which, while common worldwide, often remains undiagnosed. Depression is indeed a widespread mental health disorder. It is characterised by a depressed mood, psychomotor slowing or agitation, lack of pleasure, and inhibited expression. Depression is also accompanied by loss of appetite, weight loss, insomnia or excessive drowsiness, difficulty concentrating, crying, and suicidal thoughts [8, 12, 13]. Symptoms of depression are often classified as internalizing disorders [12, 20, 21]. In epidemiological studies, it is estimated that this disorder affects up to 15% of adolescents and young adults and is twice as likely to affect females than males. The first episodes of depression usually occur during adolescence, between 12 and 18 years of age [22]. As such, mental health issues are relatively common in the general youth population; however, there is an overall consensus that youth involved in the juvenile justice system show high rates of mental health issues [23, 24]. Research conducted among groups of juvenile offenders shows that they typically experience serious mental health problems, including depression [23, 25]. Teplin and colleagues pointed out that among adolescents, two-thirds of boys and nearly three-quarters of girls meet the diagnostic criteria for one or more psychiatric disorders [26]. Shrier and colleagues pointed out that depressive symptoms among adolescents often serve as predictors for, among others, risky sexual behaviours (e.g., not using condoms) and, as a result, sexually transmitted infections [27]. Researchers who undertake to study youth who show symptoms of demoralisation and commit criminal acts typically focus on the causes of their inability to adapt. When investigating individual and environmental links to juvenile delinquency, they often focus on risk factors. Some widely researched risk factors leading to juvenile crimes include—among others—age [28]; sex [29, 30]; neurological deficits [31, 32]; low IQ [33, 34]; physiological and genetic characteristics [35]; personality traits [36], including impulsiveness [32]; heartlessness and emotional deprivation [37]; information-processing deficits and negative attribution bias [38, 39]; emotional and evasive coping strategies [40, 41] strict parenting [42]; poor anger management skills and interpersonal competences [43, 44]; peer rejection [45, 46]; and domestic and community violence [46–48]. It is worth pointing out that the analysis of juvenile resources is a marginal issue. The number of studies on the mental health of juvenile offenders, [18, 23, 33, 36, 48, 49] however, is limited and such research has typically focused on negative indicators. The research presented herein is an attempt to fill this gap, and thus, we included to the analysis two positive mental health indicators: mental well-being and life satisfaction.

Mental well-being may be defined as an effect of the cognitive and emotional assessment of one's own life, consisting of a high level of fulfilment in multiple areas. It is one aspect of general well-being, which includes physical and social aspects of well-being [50, 51]. Mental well-being is divided into two dimensions: the first includes states of happiness and life satisfaction (hedonic dimension), and the second includes positive psychological functioning, good relations with others and self-realisation/acceptance (eudaimonic dimension) [51]. Mental well-being goes beyond hedonism and the pursuit of happiness or pleasurable experience and beyond life satisfaction: it encompasses how well people are functioning, known as eudaimonia [52]. In turn, life satisfaction is defined as a global assessment of an individual's quality of life based on the criteria chosen by that person [53]. Diener and colleagues explain it as an individual's assessment of their own life, and the more consistent both aspects are, the higher it is. Life satisfaction is therefore the result of comparing one's life situation with one's own standards [54]. Research shows that adolescents generally rate their well-being and life satisfaction as high [3, 55–60]. It should be emphasized that studies focusing on the positive aspects of mental health in groups of juveniles have not yet been conducted and the present study is an attempt to fill this gap.

A significant role in determining one's mental health is resilience. Resilience might be seen as a personality trait—a positive, distinct feature of an individual that mitigates the negative effects of stress and minimises episodes of depression [13, 61]. Resilience has also been conceptualised as a process that encompasses positive adaptation within the context of adversity [62]. Resilience is also recognised as a competence, as the capacity to handle significant changes and to assume responsibility by rebounding from adversity, uncertainty, and negativity or even positive changes. It is a positive, developable capacity that changes over time [63–65]. In this study, we understand resilience as a relatively stable personal resource, being a positive personality characteristic that can be activated or used as a personal competence and acceptance of oneself and one’s life, all of which facilitate personal adaptation, i.e., coping with change or misfortune [66, 67]. Studies have shown that resilience increases well-being and life satisfaction [67–69], eliminates the symptoms of generalised anxiety and depression and increases self-esteem, gratitude, optimism, and mental well-being [70–73]. Resilience also increases self-discipline [62, 74]. Similar relationships have also been found in youth groups. Studies show that resilience promotes well-being and life satisfaction in young people [75–77]. Young people who show high levels of resilience have been found to have fewer mental health problems [78]. Researchers [79–82] have pointed out that strengthening resilience reduces the risk of psycho-emotional and behavioural problems among adolescents. It should be emphasised that studies of the importance of resilience during adolescence rarely include juveniles who have come into contact with the law due to criminality. At this point, it is worth mentioning studies conducted by Gibson and Clarbour, who tested the structure of the Resiliency Scales for Children and Adolescents (RSCA; [83, 84]) taking into consideration its applicability in the assessment of resilience in juvenile male offenders [85]. In turn, Mowder and colleagues focused on identifying the resilience profiles of juvenile offenders of both sexes who were admitted to correctional facilities. Researchers consider the identified types of resilience to be an important resource that helps mitigate the risk factors for juveniles and prevent recidivism [86]. Kendziora and Osher focused on factors supporting resilience and the mental health of juveniles in relation to the following aspects: the family environment, local communities, systemic solutions, and the justice system [87].

Resilience correlates with a state of well-being not only directly but also indirectly through its impact on the ability to cope with stress. Resilience aids in adaptive coping. It makes it easier to mobilise oneself to take useful actions in stressful situations [88–91]. Research [92–96] has demonstrated a positive link between resilience and task-based stress coping. Task-based strategies focus on efforts targeted towards a source of stress and are aimed at changing a person, an environment, or a relationship between the two; such strategies include planned problem solving, positive revaluation, active coping strategies, or confrontation [97–100]. In addition, problem-focused strategies promote mental health [101, 102]. Studies have also shown that strategies focused on emotion, such as denial/avoidance, distraction or minimisation, finding meaning, self-blaming, and discharge/sharing feelings are negatively correlated with resilience, life satisfaction, positive affect and positively with depression, and anxiety [4, 92, 95].

We conducted two studies. The aim of the first study was to analyse the relationship between resilience and negative (depression risk assessment) and positive (life satisfaction) mental health indicators among juveniles admitted to youth education centres. Based on the results of previous studies, we expected a significant negative correlation between resilience and depression risk assessment and a positive correlation between resilience and life satisfaction among juveniles [40, 68, 69]. The aim of this study was also to analyse differences in resilience, depression risk assessment and sex differences in life satisfaction. In another study (Study 2), we expanded the scope of analysis to include relationships that combine resilience, coping strategies, and the mental health (mental well-being) of juveniles. Study 2 aimed to understand the direct and indirect role of resilience in shaping the mental health of juveniles. In the investigation, we focused on a positive indicator of mental health: mental well-being. In the model we tested, we looked at the relationship among resilience, coping strategies, and mental well-being.

## Method

### Study 1

#### Participants and procedure

The research included 201 juveniles, 60% of whom were boys (*n* = 121) and 40% who were girls (*n* = 80) between 13 and 18 years of age (*M* = 15.71; *SD* = 1.29. They were juveniles sent to youth educational centres throughout Poland. People admitted to such places are youngsters who are demoralised or have committed punishable acts and are 13–18 years of age. In each case, a family court (as an educational measure) placed the offender in a youth educational centre. During the study period, there were approximately 5000 juveniles in 94 centres across Poland [103–106].

The research was carried out on the basis of direct contact with the respondents and over the Internet. Some people received a printed copy of the research survey directly from the researcher, who explained the purpose of the research and discussed the voluntary nature of participation. In this regard, it was made clear that each person could cease to participate in the research at any point. Respondents who participated over the Internet received an email with the abovementioned information. A link to the survey was sent to the directors of the centres. Next, the directors of the facility sent an e-mail with a password and a link to the charges who expressed willingness to participate in the study. The respondents filled out, first, a scale used to measure resilience, then the scale of depression risk assessment and, finally, the scale for measuring life satisfaction. All participants gave written consent in compliance with the Helsinki Declaration. The research project was approved by the Ethics Committee of the Institute of Psychology, Polish Academy of Sciences Edition No. 22/XII/2018. Data were collected from January 2019 to June 2019.

#### Measures

To measure life satisfaction, we used the Polish adaptation [107] of the Satisfaction with Life Scale (SWLS; [54]). The scale captures global cognitive satisfaction with one's life. It consists of five statements to which responses are given using a 7-point Likert-type scale ranging from 1 (I totally disagree) to 7 (I totally agree). Item scores are summed to yield an overall measure of satisfaction with life; higher scores indicate greater satisfaction. The Polish version of the SWLS was shown to have an internal reliability of α = 0.81 [107].

To measure depression, we applied the short version of the Kutcher Adolescent Depression Scale (KADS; [108]), an instrument commonly used to screen for the risk of depression amongst young people. The scale consists of six items referring to (1) sadness; (2) lack of self-confidence; (3) physical exhaustion; (4) the belief that life is difficult and overwhelming; (5) anxiety; and (6) emerging suicidal thoughts and plans. Respondents use a 0–3 scale to indicate how frequently they experience each emotion, belief, or state (0: rarely; 1: sometimes; 2: often; 3: always). Scores of 6 or more indicate that the respondent is at risk for depression. The reliability (expressed as Cronbach’s alpha) of the Polish version was found to be α = 0.82 [22].

Resilience was measured by the Resilience Scale (RS-14; [67]), and the Polish adaptation was developed by Surzykiewicz and colleagues. The scale consists of 14 items. The respondents were asked to rate the degree to which they agree or disagree with each item. All items were assessed on a 7-point scale from 1 ('I do not agree') to 7 ('I agree'). The Polish version of the RS-14 has shown very good test–retest reliability (0.88) and good internal consistency (α = 0.85; [109]).

#### Statistical analysis

An a priori G*Power 3.1. [110] analysis was conducted to determine the suitable sample size. We used the suggested higher power criteria of 0.95 and a critical significance level of α of 0.05 to identify a medium effect size of f^2^ = 0.15. The total number of variables is 4. G*Power analysis with the abovementioned parameters would demand a sample of at least 129 participants.

Student’s t-test was used to assess potential group differences in resilience, depression risk assessment and life satisfaction. Cohen's d statistics were used to determine the effect size. Values ranging from 0.2 to 0.5 were interpreted as small, those ranging from 0.5 to 0.8 were interpreted as medium, and those above 0.8 were interpreted as large. Pearson's r correlation coefficient was used to determine the relationship between the variables, which made it possible to establish the strength and shape of the linear relationship between two variables. Values between 0 and 0.3 were interpreted as a weak correlation; from 0.3 to 0.5, moderate; from 0.5 to 0.7, strong; and from 0.7 to 1, very strong [111]. The determinants (predictors) of depression risk assessment and life satisfaction were identified on the basis of hierarchical regression analysis. A variable that explained at least 5% of the total variance of the dependent variable was considered to be a predictor. The significance of the linear regression coefficients was checked using Student's t-test, which tests the null hypothesis and shows that the given coefficient does not differ significantly from zero.

### Results

#### Descriptive results

Table 1 shows the averages and standard deviations of two mental health indicators: risk assessments of depression and life satisfaction and resilience across the entire group of juveniles studied (and separately among girls and boys). The mean life satisfaction rate in the study group was *M* = 19.36 (*SD* = 7.20). The results pertaining to juveniles show an average level of life satisfaction (5–6 stanines, raw score 18–23 pts.) [112]. Life satisfaction scores varied between adolescent girls and adolescent boys: adolescent girls (*M* = 16.20, *SD* = 7.26) showed significantly lower life satisfaction levels than their male counterparts (*M* = 21.45, *SD* = 6.37). The effect of differences in life satisfaction can be defined as average, with Cohen's *d* = 0.77. The average intensity of the risk of depression in the examined group was *M* = 5.95 (*SD* = 4.62). On the Polish adaptation of the KADS [22], a score equal to or higher than 6 points is considered to indicate a risk of depression. No significant differences were found between girls and boys with respect to the depression risk score. The average resilience score in the study group overall was *M* = 67.66 (*SD* = 15.15). The results obtained on the resilience scale were within the range of 4–5 stanines and can be viewed as average scores [109]. The results differed significantly between the adolescent girls *M* = 63.47 (*SD* = 14.09) and adolescent boys *M* = 70.43 (*SD* = 15.24): girls had significantly lower levels of resilience than adolescent boys. Differences in the case of the level of resilience can be described as having a low effect size, with average Cohen's *d* = 0.47.

**Table 1:** Descriptive statistics and differences in life satisfaction, depression risk assessment and resilience in the entire group and in the group of juvenile girls and juvenile boys

	Whole group		Juvenile boys		Juvenile girls		*t*
	*M*	*SD*	*M*	*SD*	*M*	*SD*	
Life satisfaction	19.36	7.20	21.45	6.37	16.20	7.26	− 5.40*
Depression risk assessment	5.95	4.62	5.66	4.63	6.38	4.59	1.09
Resilience	67.66	15.15	70.43	15.24	63.47	14.09	− 3.31*

*p < .001

#### Resilience and mental health (risk of depression and satisfaction with life)

Correlation analysis showed that resilience was positively and significantly related to life satisfaction (*R* = 0.65) and negatively and moderately related to depression risk assessment (*R* = − 0.31). The next stage of the statistical analysis was to determine the predictors used in the study of mental health indicators. For this purpose, linear regression analysis with a hierarchical input method was used. The first model included sex, the second added resilience, and the third model included the interaction of sex and resilience. Prior to the analysis, the variables were standardised, and the moderator was centred (+ 1 girls, − 1 boys). Table 2 presents the results of hierarchical regression analysis.

**Table 2:** Results of hierarchical regression analysis with sex as a moderator

Variable	Life satisfaction						Depression risk assessment					
	B	*SE B*	Beta	t	*p*	∆*R*^2^	B	*SE B*	Beta	t	*p*	∆*R*^2^
STEP 1												
Resilience	.65	.05	.65	12.11	.001	.42*	− .31	.07	− .31	− 4.55	.001	.09*
STEP 2												
Resilience	.60	.05	.60	11.33	.001		− .31	.07	− .31	− 4.39	.001	
Sex	− .23	.05	− .22	− 4.19	.001	.05*	.01	.07	.01	.12	ns	.00
STEP 3												
Resilience	.63	.06	.63	11.46	.001		− .33	.07	− .33	− 4.60	.001	
Sex	− .22	.05	− .21	− 3.98	.001		− .002	.07	− .002	− .03	ns	
Resilience x sex	.10	.06	.10	1.85	.07	.01	− .10	.07	− .01	− 1.33	ns	.01

*ns* not significant

Given that the third model explained the largest percentage of variance in life satisfaction, it was adopted as the final model for interpretation. This model proved to be well suited to the data *F*(3.197) = 60.66, *p* < 0.001. Regression analysis showed that significant predictors of life satisfaction were resilience (β = 0.63) and sex (β = − 0.21). The effect of the interaction of both variables was nonsignificant. The variables included in the model explained 47% of the variability of the dependent variable. Additionally, in the case of depression risk assessment, the third model explained the largest percentage of the explained variable. The model was well fitted to the data *F*(3.197) = 7.48, *p* < 0.001. In this model, the only significant predictor of depression was resilience (β = − 0.33), which explained 9% of the variability of the dependent variable (Table 2).

### Study 2

#### Participants and procedure

Study 2 involved juveniles admitted to youth educational centres throughout Poland. The study included 253 juveniles, 68% of whom were boys (*n* = 172) and 32% who were girls (*n* = 81) between 13 and 18 years of age (*M* = 16.34, *SD* = 1.16). The study was carried out through direct contact with respondents. Respondents received a printed copy of the research survey directly from the researcher who explained the purpose of the research. The researchers also advised the young people on the voluntary nature of participation and emphasised that each person could cease participating in the research at any point. All participants gave written consent in compliance with the Helsinki Declaration. The research project was approved by the Ethics Committee of the Institute of Psychology, Polish Academy of Sciences Edition No. 22/XII/2018. The respondents first filled out the scale used to measure resilience, then the questionnaire concerning stress coping, and finally the scale to measure mental well-being. Data were collected from September 2019 to December 2019.

#### Measures

*Mental well-being* Mental well-being was measured using the Warwick-Edinburgh Mental Well-being Scale (WEMWBS; [50], Polish adaptation by Authors et al. (in review)). The scale consists of 14 items. Over a 2-week period, at intervals, respondents rate the intensity of their own experiences and emotions on a 5-point Likert-type scale where 1 is 'never' and 5 is 'always'. In Polish research, the reliability of the scale calculated using Cronbach's alpha turned out to be high (α = 0.92).

*Resilience* Similar to Study 1, resilience was measured by the Resilience Scale—RS-14 [67], with the Polish adaptation by Surzykiewicz and colleagues [109].

*Strategies for dealing with stress* The Brief-COPE Questionnaire [113] was administered to measure coping strategies. The scale consists of 28 statements (2 statements about each of 14 strategies). Responses are given using a 4-point Likert-type scale (0: 'I almost never do that'; 1: 'I rarely do that; 2: 'I often do that'; 3: 'I almost always do that'). In the Polish version of the scale, Cronbach's alpha coefficients for the individual strategies were found to range from 0.48 to 0.94 [112, 113]. The results of CFA confirmed the seven-factor model of the Brief-COPE Questionnaire, and thus, seven coping strategies were identified: (1) active, positive coping (positive re-evaluation, acceptance, active coping and planning); (2) seeking support from others (of instrumental and emotional nature); (3) coping by discharging negative emotions (discharge and denial); (4) shifting towards religion; (5) coping through humour; (6) coping through avoidance; and (7) coping through the use of alcohol and psychoactive substances.

#### Statistical analyses

Similar to Study 1, an a priori G*Power 3.1. [110] analysis was conducted to identify a suitable sample size. We used the suggested higher power criteria of 0.95 and a critical significance level of α of 0.05 to identify a medium effect size of f^2^ = 0.15. The total number of variables is 9. G*Power analysis with the abovementioned parameters would demand a sample of at least 160 participants.

Pearson's r correlation coefficient was used to determine the relationship between the variables. Structural equation modelling was used to verify the hypotheses. Analysis of structural equations was carried out with the use of the AMOS program. Model parameters were estimated with the use of the maximum likelihood method. To assess the accuracy of the fit of the model to the data, the following indicators were used: CFI (confirmatory fit index), GFI (goodness-of-fit index), RMSEA (root-mean-square error of approximation) and relative chi-square (χ^2^/df) [114, 115]. GFI ≥ 0.90 and CFI ≥ 0.95 indicate that a model has a good and adequate fit to the data [116, 117]. Values of χ^2^/df < 2 also suggest a good fit of the model to the data [115, 116]. RMSEA < 0.08 can also be interpreted as a good fit to data [118].

### Results

#### Descriptive results

Table 3 presents the means and standard deviations of the variables as well as the correlation matrix for the relationship among resilience, coping strategies, and well-being. A significant relationship between resilience and mental well-being was observed. Interrelationships between stress coping strategies and resilience were also reported. Resilience was positively associated with active coping, seeking support from others, avoiding, and coping by shifting towards religion. In contrast, negative relationships were observed between resilience and both coping by discharging negative emotions and using psychoactive substances. No relationship between resilience and humour-related strategy was observed. There were also significant relationships between coping strategies and well-being. Active coping, seeking support from others, avoiding and turning towards religion were all positively correlated with well-being, while coping by discharging negative emotions and using psychoactive substances was negatively correlated. No significant relationship between well-being and humour was observed.

**Table 3:** Correlations, means and standard deviations for measured variables

Variables	1	2	3	4	5	6	7	8	9
1. Mental well-being	1								
2. Resilience	.71**	1							
3. Active coping	.56**	.64**	1						
4. Seeking support	.54**	.44**	.60**	1					
5. Avoidance coping	.27**	.21**	.43**	.35**	1				
6. Emotion coping	− .23**	− .22**	.05	.00	.27**	1			
7. Substance use	− .25**	− .17**	− .20**	− .21**	.09	.33**	1		
8. Religion	.15*	.18**	.28**	.12	.30**	.14*	.03	1	
9. Humor	.07	.11	.02	− .06	.19**	.13*	.24**	.22**	1
M	51.94	72.88	2.02	1.99	1.67	1.49	1.06	1.04	1.25
*SD*	10.45	14.99	.65	.72	.64	.75	.99	.03	.89

*p < .05; ** *p* < .01; N = 253; *M* mean; *SD* standard deviation

#### Resilience, coping strategies and mental well-being

**Fig. 1:**
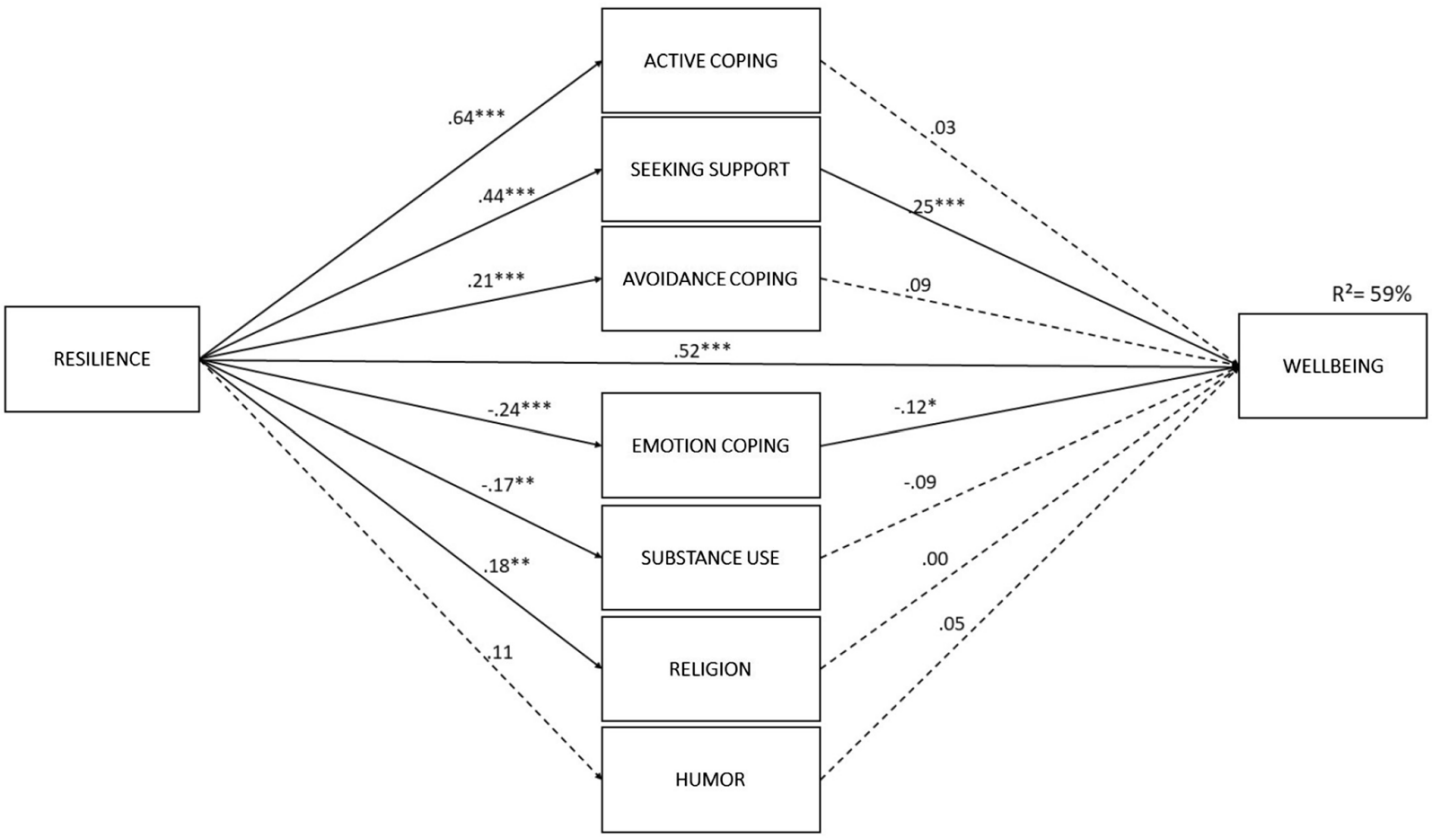
Predictors of mental well-being in a group of juveniles (N = 253, * *p* < .05; ** *p* < .01; *** *p* < .001)

As a next step, structural equation modelling was used to analyse the relationship among resilience, stress coping strategies, and mental well-being. The model includes correlations of errors between areas of stress management strategies. The model turned out to be well suited to the data, χ^2^(6) = 11.71, *p* = 0.06, χ2/*df* = 1.95, RMSEA = 0.061 (low = 0.000; high = 0.114), GFI = 0.99; CFI = 0.99. Resilience was an important direct predictor of mental well-being (β = 0.52, *p* < 0.001). Analysis of path values showed that most of the relationships between resilience and coping strategies were statistically significant. Resilience was an especially important predictor of active coping (β = . 64, *p* < 0.001), seeking support from others (β = 0.44, *p* < 0.001) and, to a lesser extent, avoidance strategies (β = 0.21, *p* < 0.001). Resilience was negatively associated with coping by discharging negative emotions (β = − 0.24, *p* < 0.001) and the use of psychoactive substances (β = − 0.17, *p* < 0.01). Significant but weaker relationships were observed between resilience and coping through religion (β = 0.18, *p* < 0.01). No relationship between resilience and humour was observed (β = 0.11, *p* > 0.05). In addition, seeking support from others and coping through discharging negative emotions mediated the relationship between resilience and mental well-being. The analysis showed a significant indirect effect of strategies focused on the discharge of negative emotions (β = 0.03; p < 0.01) and seeking support from others (β = 0.11; p < 0.001). Taking into account indirect effects, the total impact of resilience on mental well-being was 0.52. Overall, resilience and coping strategies accounted for 59% of the variance in mental well-being (Fig. 1).

## Discussion

The presented research focuses on the role of resilience in the mental health of juveniles and we conducted two separate studies. Study 1 demonstrated a significant, positive relationship between resilience and life satisfaction. In line with earlier findings, the stronger the degree of resilience, the greater the life satisfaction [57, 58, 119–121]. The results also showed that high levels of resilience can act as a buffer that protects juveniles against the risk of depression. This finding is consistent with previous literature exploring the influence of resilience on mental health problems among adolescents [12–14, 49, 78, 81, 82, 93, 122, 123]. Moreover, the results showed significant differences in levels of resilience and life satisfaction in the group of juvenile girls and boys. However, in contrast to other findings in youth groups demonstrating no sex difference in resilience, [66, 124] the girls in the group of juveniles had a significantly lower level of resilience and life satisfaction than the boys. No significant differences were found in the depression risk assessment for juvenile girls and boys. In addition, there was no significant interaction effect between life satisfaction and sex or between depression risk assessment and sex.

The results of Study 2 have shown that resilience is an important and positive predictor of the mental well-being of juveniles and these results are consistent with earlier studies of youth groups [3, 72, 125–129]. In addition, resilience not only had a direct impact on the mental well-being of juveniles but also had an indirect impact through its relationship with two coping strategies: seeking support from others and coping through emotions. Both of these strategies mediated the relationship between resilience and mental well-being. At the same time, relationships among resilience, seeking support, and mental well-being were positive; those among resilience, coping through emotions, and mental well-being were negative. Furthermore, the indirect effect of seeking support from others on mental well-being was stronger than using strategies focused on the discharge of negative emotions. This means that resilience reduces the tendency of juveniles to focus on the negative emotions experienced and the need to discharge them but also diminishes their propensity to believe they should deal with problems on their own, without relatives, colleagues and professionals, regardless of the circumstances. In other words, resilience intensifies the tendency to cope using strategies related to seeking support. The psychological and material resources provided by a social network help juveniles to cope with difficult situations. In turn, the sense of belonging, intimacy and helpful advice all serve to improve the mental well-being of juveniles, along with a reduced need to discharge negative emotions.

In the Study 2 significant relationships were observed between resilience and active, positive coping, turning to religion, coping through avoidance, and coping by using psychoactive substances; however, none of these strategies was a significant direct predictor of mental well-being. Furthermore, a significant positive relationship was noted between resilience and avoidance coping, as well as a positive but not significant path from avoidance coping to mental well-being. Although avoidance coping is known as a maladaptive avoidance strategy, some forms of this strategy might actually be healthy [92, 93]. Contrary to avoidance behaviours such as stopping, distracting, or withdrawing, avoidance coping related to seeking contact with others, reading books, or doing work can be adaptive [94, 130]. These healthier forms of coping do not necessarily approach the problem directly, but they do affect the response to the problem [131]. To summarise, the results obtained confirm the results of previous studies demonstrating the significant relationship between resilience and coping strategies [92, 95, 96, 101, 132].

The study found that resilience intensifies the tendency to cope using strategies related to seeking support, which has a direct impact on the mental well-being of socially maladjusted juveniles admitted to youth educational centres. These findings are important for educational work with this group of young people, especially the girls, who had a significantly lower level of resilience than the boys. Resilience is conducive to seeking support from other people, e.g., peers, teachers, or educators. Such actions enable juveniles to deal with burdens and challenges more effectively and, in the end, to feel greater satisfaction and find greater meaning in life. Resilience also reduces their tendency to focus on negative emotions experienced and the need to discharge them—the latter strategy can threaten their mental well-being.

Our research confirms that resilience and specific coping strategies are resources that help maladapted adolescents achieve life satisfaction and well-being—both aspects of mental health in its broad sense. For this reason, educational activities aimed at strengthening resilience and shaping coping skills in groups of maladjusted youth are important. In the creation of programmes targeting youth, it is worth shifting the focus from deficits and dysfunctions (risk factors) to resources (protective factors) and seeing juveniles less as a group characterised by specific risk factors but more as a group needing resources that must be developed [133–135]. When designing preventive and resocialisation programmes, in order to achieve a full understanding of how young people function, their ability to achieve both successful and problematic results in their development should be taken into account. Similarly, intervention programmes should cover a full range of experiences and opportunities for young people in this period of development. With respect to development, such activities must take into account a natural context for this period and consider the changes in the types of adverse situations and competences needed to deal with them. Therefore, to keep up with these changing needs and situations, it is important to adapt such a programme to the development stage. Moreover, protective factors work at different levels. For actions to be realistic and interventions to be effective, it is worth considering how individual capabilities affect external protective factors. Therefore, there is a need for further research on the interaction of adversities, internal and external resilience resources, and appropriate interventions.

The results obtained indicate that work on the level of juvenile resilience leads to the strengthening of positive mental health indicators and buffering of negative indicators. Therefore, we believe that for remedial and resocialisation measures to work effectively, the functioning of individuals should be analysed not only in the context of factors leading to social maladjustment but also particularly from the perspective of the resources they have. Paradoxically, we know less about the factors that can help juveniles than about the factors that lead to maladjustment. Hence, the first action that an educator in a social rehabilitation institution should take is to diagnose and identify a wide range of juveniles, followed by the development of an appropriate intervention programme based on the identified resources. The use of a research framework in the field of resilience can help interested practitioners build intervention programmes focused on creating and strengthening the resources and assets of juveniles.

The studies conducted have some limitations that should be taken into account when drawing practical conclusions and preparing further research. In Study 1, the explained variance in the risk of depression was only 9%; i.e., while significant, this figure is not high. This result occurred because only one predictive variable was included—i.e., resilience—which simultaneously, together with sex, accounted for a significantly higher percentage of the variance in life satisfaction (47%). Further research should consider other juvenile resources that may affect their mental health, such as toughness, resilience, self-efficacy, or social resources. In addition, the level of social maladjustment was not analysed in this study, and this variable could moderate the relationship between the analysed variables. Thus, further research should identify differences in the level of mental health of juvenile groups in terms of the level of social maladjustment while looking for an array of resources conducive to promoting mental health in these groups. The second study did not analyse the negative indicator. It might be useful to explore the mediating role of coping strategies in the relationship between resilience and depression risk assessment in the group of juveniles.

## Conclusions

In conclusion, the results of the research show that resilience increases life satisfaction and mental well-being and minimises depression risk assessment for juveniles. Stress coping strategies also play a mediating role in the relationship between resilience and mental well-being. This study was one of the first to focus on two-dimensional perceptions of mental health and the assessment of relationships between resilience, coping strategies, and mental health indicators in a group of juveniles.

## Data Availability

The data that support the findings of this study are available from: https://doi.org/10.3886/E120001V1

## Abbreviations

CFI: Confirmatory fit index
GFI: Goodness-of-fit index
RMSEA: Root-mean-square error of approximation
KADS: Kutcher Adolescent Depression Scale
WEMWBS: Warwick–Edinburgh Mental Well-being Scale
RS: Resilience Scale
SWLS: Satisfaction with Life Scale
